# Augmentation of protective immune responses against viral infection by oral administration of schizophyllan

**DOI:** 10.1080/09629359791596

**Published:** 1997-11

**Authors:** Wataru Itoh

**Affiliations:** 1 Research Laboratory Taito Corporation Ltd 1–26 Higashi-shiriike-shinmachi Nagata-ku Kobe-shi Hyogo 653 Japan

## Abstract

An oral administration of fungal polysaccharide schizophyllan has augmented protective immune responses to Sendai virus infection in mice and the rodshaped DNA virus of *Penaeus japonicus* (RV-PJ) infection in Kuruma shrimps. When schizophyllan was administered orally at a dose of 50 or 100 mg/kg body weight per day, the survival rates after virus challenge were significantly higher than those of the control groups. High phagocytic activities were observed in the haemocytes of the schizophyllan-fed shrimps.These results suggest that schizophyllan confers effective protection against viral infection by increasing antiviral immune responses, and that it could be used to boost immunity to virus infection in animals or in invertebrates.


Mediators of Inflammation, 6, 267–269 (1997)

### Augmentation of protective immune responses against viral infection by oral administration of schizophyllan

**Wataru Itoh**

Research Laboratory, Taito Corporation Ltd, 1–26 Higashi-shiriike-shinmachi, Nagata-ku, Kobe-shi, Hyogo 653, Japan

Tel: +81 78 651 5408
Fax: +81 78 651 5425

**An oral administration of fungal polysaccharide schizophyllan has augmented protective immune responses to Sendai virus infection in mice and the rod-shaped DNA virus of *Penaeus japonicus* (RV-PJ) infection in Kuruma shrimps. When schizophyllan was administered orally at a dose of 50 or 100 mg/kg body weight per day, the survival rates after virus challenge were significantly higher than those of the control groups. High phagocytic activities were observed in the haemocytes of the schizophyllan-fed shrimps. These results suggest that schizophyllan confers effective protection against viral infection by increasing antiviral immune responses, and that it could be used to boost immunity to virus infection in animals or in invertebrates.**

**Keywords:** Immune response, animal experiments, protective factors

### Introduction

Schizophyllan is a water-soluble, non-ionic polysaccharide produced extracellularly by the fungus *Schizophyllum commune* Fries. It consists of a main chain of (1→3)-β-D-glucose residues substituted at 0–6 by a single-unit β-D-glucosyl residue, with a side chain of a β-1,6-D-glucosyl group at every three glucose residues of the main chain [1]. Schizophyllan has antitumor and antimicrobial activities, mediated by stimulation of the host immune systems [2,3]. Schizophyllan is well known in Japan as a biological response modifier in clinical use [4].

It has been reported that schizophyllan activates host-defense systems such as macrophages, natural killer cells, antibody-dependent cellular cytotoxicity and cytotoxic T lymphocytes. These immune systems also provide an important level of protection against viral infection [5,6]. Therefore, schizophyllan has potential use as an agent to increase protective immunity against viral infection.

Recently, infectious viral diseases have occurred frequently worldwide in culture farms of some animals, fishes and shrimps. In Japan, a new acute viral disease was identified in 1993 at shrimp farms and has caused severe losses to shrimp farmers. The causative agent was a rod-shaped DNA virus of *Penaeus japonicus* (RV-PJ) [7].

Here we present data on the oral administration of schizophyllan and the immune responses to Sendai virus infection in mice and RV-PJ infection in shrimps.

### Materials and methods

### Schizophyllan

Schizophyllan with a molecular weight of $4.6 \times 10^5$ was used for the Sendai virus challenge test, and crude schizophyllan for the shrimp test. Crude schizophyllan is a dried culture broth of the schizophyllan-producing fungus *Schizophyllum commune* Fries. Both substances were manufactured by Taito Co., Ltd (Kobe, Japan).

### Sendai virus challenge test in mice

Specific-pathogen-free, 3-week-old male Institute of Cancer Research/Charles River Japan Inc. (ICR/CRJ; CD-1) mice were treated orally with schizophyllan (100 mg/kg body weight per day) by a feeding tube for 5 days. Control mice were given the same volume of phosphate-buffered saline instead of schizophyllan. The mice were inoculated intranasally with $4.0 \times 10^6$ cell infectious units (10 times the median lethal dose, $LD_{50}$) of the Fushimi strain of the Sendai virus. After virus inoculation, the mice were further treated with schizophyllan for an additional 2 days. The protective effects of schizophyllan were assessed on the basis of survival ratios for the test mice [8]. A protocol of the test is shown in Fig. 1.

### RV-PJ challenge test in shrimps

Two groups of Kuruma shrimps (10–20 g), with 30 shrimps in each group, were given none or 50 mg

© 1997 Rapid Science Publishers

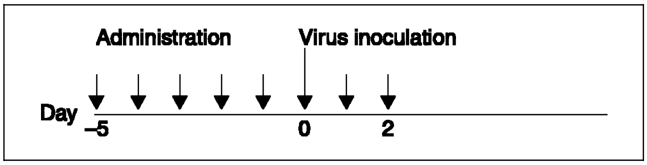

FIG. 1. Protocol of Sendai virus-challenge test.

crude schizophyllan/kg body weight/day in their diet for 7 days. Then the shrimps were challenged by intramuscular injection with 0.1 ml extracted filtrate from lymphoid tissues of diseased shrimps.

### Phagocytic activity

Hemocytes were obtained from the hearts of shrimps. The hemocytes ($1 \times 10^5$ cells/ml) were mixed with fluorescence-labeled latex beads ($1 \times 10^8$ beads/ml) in K199 medium [9]. After incubation at 25°C for 30 min, the number of ingested beads and number of phagocytizing cells were counted from any 200 cells observed. The phagocytic index was calculated as: (no. of cells ingesting beads/no. of cells observed) × (no. of beads ingested/no. of cells observed) × 100.

### Results

### Protection of mice by schizophyllan against Sendai virus infection

Fig. 2 shows that 50% of mice treated orally with schizophyllan survived the viral pneumonia. The survival ratio was significantly higher than that of control mice. The efficacy of the oral administration of schizophyllan was almost equal to that of intraperitoneal injection.

### Protection of shrimps by crude schizophyllan against RV-PJ infection

Fig. 3 shows changes in survival rates after a virus challenge in shrimps given food with or without crude schizophyllan. The final survival rates were 37% in crude schizophyllan-fed shrimps and 10% in controls. These results demonstrate that an oral administration of crude schizophyllan increases the disease-resistance of shrimps against RV-PJ infection 4–9 days after administration.

### Phagocytic activity

Fig. 4 shows the changes in phagocytic activity of shrimp hemocyte when crude schizophyllan was administered orally. The phagocytic activity of the crude schizophyllan-fed shrimps was significantly higher than that of controls ($P < 0.05$). These results indicate that shrimp hemocytes are activated by the oral administration of crude schizophyllan.

### Conclusions

One basic and important strategy to prevent virus infectious diseases is to increase the protective immune responses of a host. The results outlined here show

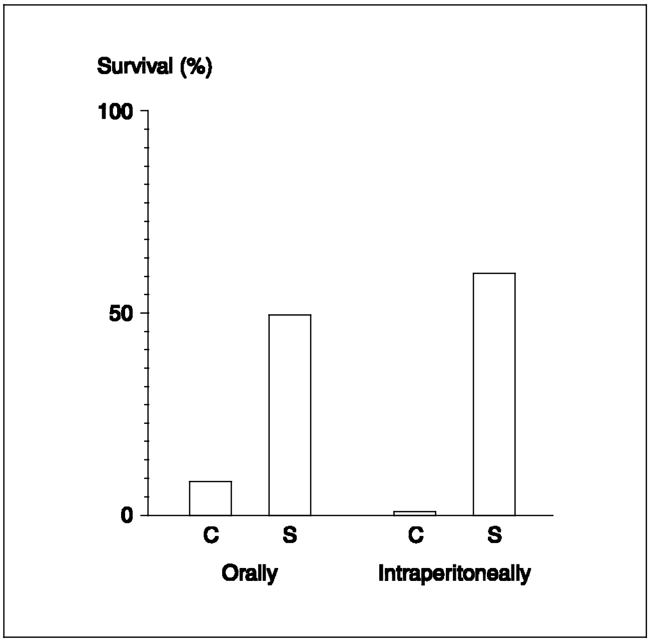

FIG. 2. Protective effect of schizophyllan (S) against Sendai virus infection in mice. C, control.

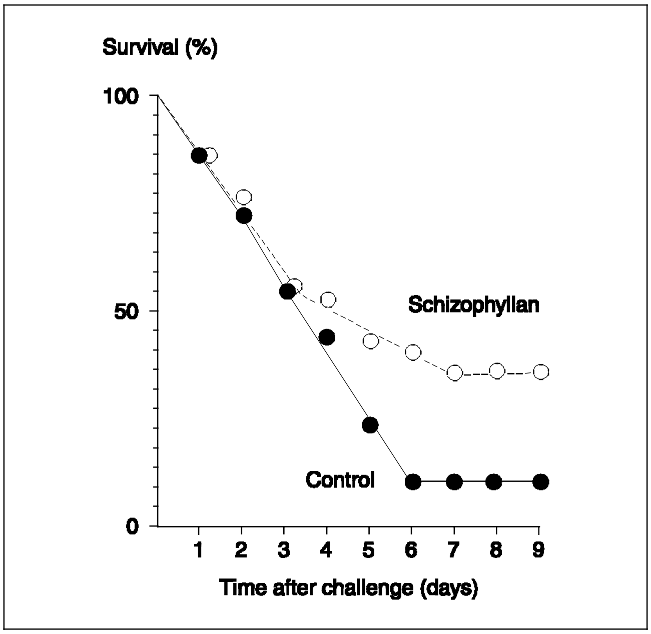

FIG. 3. Protective effect of an oral administration (50 mg/kg body weight per day) of schizophyllan against infection by the rod-shaped DNA virus of *Penaeus japonicus* (RV-PJ) in Kuruma shrimps.

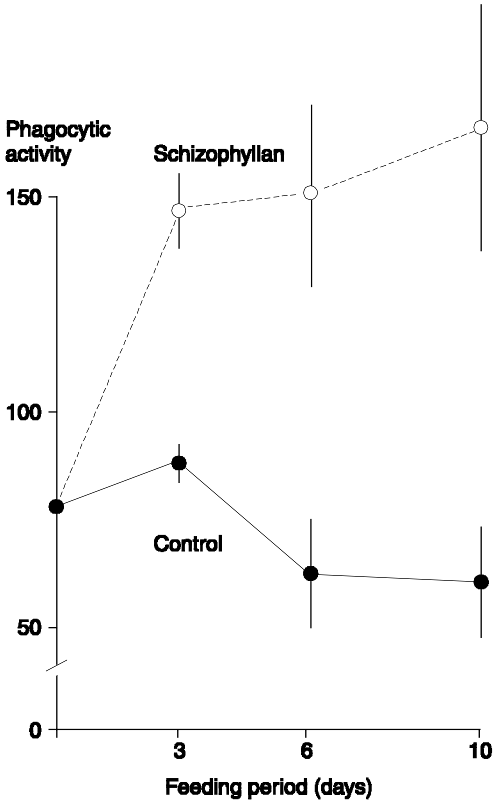

FIG. 4. Increase in phagocytic activity by shrimp hemocytes after an oral administration of schizophyllan.

that an oral dose of schizophyllan induces a protective immune response against virus infection in mice and against RV-PJ infection in shrimps. Thus schizophyllan may have potential as an immune enhancer against viral infection in animals or in invertebrates.

### References

1. Kikumoto S, Miyajima T, Yoshizumi S, Fujimoto S, Kimura K: Polysaccharide produced by schizophyllan commune: Part 1. Formation and some properties of an extracellular polysaccharide. *Nippon Nogeikagakukaishi* 1970, **44**:337–342.
2. Komatsu N, Okubo S, Kimura K, Saito G, Sakai S: Host-mediated antitumor action of schizophyllan, a glucan produced by *Schizophyllum commune*. *Jpn J Cancer Res (GANN)* 1969, **60**:137–144.
3. Komatsu N, Nagumo N, Suzuki M, Matsuo T: Effect of schizophyllan on experimental infections and tumors [abstract]. *Int J Immunopharmacol* 1980, **2**:171.
4. Okamura K, Suzuki M, Chihara T, *et al.*: Clinical evaluation of schizophyllan combined with irradiation in patients with cervical cancer. *Cancer* 1986, **58**:865–872.
5. Kikuchi M, Iwano K, Numazaki Y: Effect of schizophyllan on immune function and on its anti-tumor activity. *Int J Immunopharmacol* 1980, **2**:173–174.
6. Kikuchi M, Iwano K, Numazaki Y, Komuro K: Augmented induction of ADCC and NK cell activities by schizophyllan (SPG), an antitumor polysaccharide. *Med J Kinki Univ* 1981, **6**:375–383.
7. Inoue K, Yamano K, Ikeda N, *et al.*: The penaeid rod-shaped DNA virus (PRVD), which causes penaeid acute viremia (PAV). *Fish Pathol* 1996, **31**:39–45.
8. Hotta H, Hagiwara K, Ito W, Homma M: Augmentation of protective immune response against Sendai virus infection by fungal polysaccharide schizophyllan. *Int J Immunopharmacol* 1993, **15**:55–60.
9. Itami T, Takahashi Y, Tsuchihira E, Igusa H, Kondo M: Enhancement of disease resistance of Kuruma prawn *Penaeus japonicus* and increase in phagocytic activity of prawn hemocytes after oral administration of β-1,3-glucan (schizophyllan). In: *The Third Asian Fisheries Forum.* Edited by Chou LM, Munro AD, Lam TJ, *et al.* Manila: Asian Fisheries Society; 1994:375–378.

